# The European KIDSCREEN approach to measure quality of life and well-being in children: development, current application, and future advances

**DOI:** 10.1007/s11136-013-0428-3

**Published:** 2013-05-18

**Authors:** Ulrike Ravens-Sieberer, Michael Herdman, Janine Devine, Christiane Otto, Monika Bullinger, Matthias Rose, Fionna Klasen

**Affiliations:** 1 Department of Child and Adolescent Psychiatry, Psychotherapy, and Psychosomatics, University Medical Center Hamburg-Eppendorf, Martinistr. 52 (W29), 20246 Hamburg, Germany; 2 Insight Consulting and Research, Cami Ral 266, 08301 Mataro, Barcelona Spain; 3 Department of Medical Psychology, University Medical Center Hamburg-Eppendorf, Martinistr. 52, 20246 Hamburg, Germany; 4 Department for Internal Medicine and Psychosomatics, Charité, University Medicine, Berlin, Luisenstraße 13, 10117 Berlin, Germany

**Keywords:** Quality of life, Children, Adolescents, KIDSCREEN, Generic measurement

## Abstract

**Purpose:**

The KIDSCREEN questionnaires were developed by a collaborative effort of European pediatric researchers for use in epidemiologic public health surveys, clinical intervention studies, and research projects. The article gives an overview of the development of the tool, summarizes its extensive applications in Europe, and describes the development of a new computerized adaptive test (KIDS-CAT) based on KIDSCREEN experiences.

**Methods:**

The KIDSCREEN versions (self-report and proxy versions with 52, 27, and 10 items) were simultaneously developed in 13 different European countries to warrant cross-cultural applicability, using methods based on classical test theory (CTT: descriptive statistics, CFA and MAP, internal consistency, retest reliability measures) and item response theory (IRT: Rasch modeling, DIF analyses, etc.). The KIDS-CAT was developed (in cooperation with the US pediatric PROMIS project) based on archival data of European KIDSCREEN health surveys using IRT more extensively (IRC).

**Results:**

Research has shown that the KIDSCREEN is a reliable, valid, sensitive, and conceptually/linguistically appropriate QoL measure in 38 countries/languages by now. European and national norm data are available. New insights from KIDSCREEN studies stimulate pediatric health care. Based on KIDSCREEN, the Kids-CAT promises to facilitate a very efficient, precise, as well as reliable and valid assessment of QoL.

**Conclusions:**

The KIDSCREEN has standardized QoL measurement in Europe in children as a valid and cross-cultural comparable tool. The Kids-CAT has the potential to further advance pediatric health measurement and care via Internet application.

## Introduction

Health-related quality of life (QoL) instruments are increasingly used as outcome measures in a variety of settings, including clinical research, population health surveys, and clinical practice, and in both adult and pediatric populations. As a consequence, the number of instruments available has also increased; a 2008 review identified 30 generic and 64 disease-specific instruments available for use in children and adolescents [1].

While adult definitions of QoL can be applied to children and adolescents, other factors can affect QoL in children and adolescents [2]. Despite recent developments, instruments used to assess QoL in children and adolescents still show problems relating to international comparability and may not take into account different cultural perspectives during their construction [3]. The KIDSCREEN project was promoted by the European Union and aimed to produce self-disclosure QoL questionnaires for healthy and chronically ill children and adolescents which gave due weight to cultural issues. The KIDSCREEN project was run in parallel with the DISABKIDS project [4], which aimed to produce condition-specific questionnaires for children and adolescents with chronic health conditions. The 13 countries which participated in both projects were Austria (AT), Czech Republic (CZ), France (FR), Germany (DE), Greece (EL), Hungary (HU), Ireland (IE), the Netherlands (NL), Poland (PL), United Kingdom (UK), Spain (ES), Sweden (SE), and Switzerland (CH) [5]. The instruments were designed for use in epidemiologic public health surveys, clinical intervention studies, and research projects.

The generic KIDSCREEN QoL measure for children and adolescents is available in three versions; the original long version consists of 52 items covering ten dimensions of QoL [see Table 1; 6, 7], a 27-item version covering 5 dimensions of QoL [8, 9], and a 10-item index version [10]. The instruments are designed to be used in populations aged 8 to 18 years and both self-complete and proxy (parent) versions are available. Normative, reference values are available for all KIDSCREEN versions for 11 European countries [5]. Since their development, all three versions have been used in a variety of settings and study designs, particularly in Europe, and new initiatives are also underway, including work on a computer-adaptive version of the instrument.

**Table 1:** Interpretation of KIDSCREEN dimensions

	Definition
KIDSCREEN-52 dimensions	
Physical well-being	This dimension explores the level of the child’s/adolescent’s physical activity, energy, and fitness. Level of physical activity is examined with reference to the child’s/adolescent’s ability to get around the home and school, and to play or do physically demanding activities such as sports, since a child’s/adolescent’s impairment does also affect physical activity. The dimension also looks at the child’s/adolescent’s capacity for lively or energetic play. In addition, the extent to which a child or adolescent feels unwell and complains of poor health is examined.
Psychological well-being	This dimension examines the psychological well-being of the child/adolescent including positive emotions and satisfaction with life. It specifically reveals the positive perceptions and emotions experienced by the individual. The questions look at how much a child/adolescent experiences positive feelings such as happiness, joy, and cheerfulness. It also reflects the person’s view of their satisfaction with life so far.
Moods and emotions	This dimension covers how much the child/adolescent experiences depressive moods and emotions and stressful feelings. It specifically reveals feelings such as loneliness, sadness, sufficiency/insufficiency, and resignation. Furthermore, this dimension takes into account how distressing these feelings are perceived to be. This dimension shows a high score in QoL if these negative feelings are rare.
Self-perception	This dimension explores the child’s/adolescent’s perception of self. It includes whether the appearance of the body is viewed positively or negatively. Body image is explored by questions concerning satisfaction with looks as well as with clothes and other personal accessories. The dimension examines how secure and satisfied the child/adolescent feels about him/herself as well as his/her appearance. This dimension is meant to reflect the value somebody assigns to him/herself and the perception of how positively others value him/her.
Autonomy	This dimension looks at the opportunity given to a child or adolescent to create his/her social and leisure time. It examines the child’s/adolescent’s level of autonomy, seen as an important developmental issue for creating an individual identity. This refers to the child’s/adolescent’s freedom of choice, self-sufficiency, and independence. In particular, the extent to which the child/adolescent feels able to shape his/her own life as well as being able to make decisions about day-to-day activities is considered. The dimension also examines whether the child/adolescent feels sufficiently provided with opportunities to participate in social activities, particularly in leisure activities and pastimes.
Parent relations and home life	This dimension examines the relationship between the parents and the atmosphere in the child’s/adolescent’s home. It explores the quality of the interaction between the child/adolescent and parent or carer, and the child’s/adolescent’s feelings toward parents/carers. Particular importance is attached to whether the child/adolescent feels loved and supported by the family, whether the atmosphere at home is comfortable or not and also if the child/adolescent feels treated fairly.
Social support and peers	This dimension examines the nature of the child’s/adolescent’s relationships with other children/adolescents. Social relations with friends and peers are considered. The dimension explores the quality of the interaction between the child/adolescent and peers as well as their perceived support. The questions examine the extent to which the child/adolescent feels accepted and supported by friends and the child’s/adolescent’s ability to form and maintain friendships. In particular, aspects concerning communication with others are considered. It also explores the extent to which the person experiences positive group feelings and how much he/she feels part of a group and respected by peers and friends.
School environment	This dimension explores a child’s/adolescent’s perception of his/her cognitive capacity, learning and concentration, and his/her feelings about school. It includes the child’s/adolescent’s satisfaction with his/her ability and performance at school. General feelings about school, such as whether school is an enjoyable place to be, are also considered. In addition, the dimension explores the child’s view of the relationship with his/her teachers. For example, questions include whether the child/adolescent gets along well with his/her teachers and whether the teachers are perceived as being interested in the student as a person.
Social acceptance (bullying)	This dimension covers the aspect of feeling rejected by peers in school. It explores both the feeling of being rejected by others as well as the feeling of anxiety toward peers. We say a student is being bullied when another student or a group of students say or do nasty and unpleasant things to him or her. It is also bullying when a student is teased repeatedly in a way he or she does not like. But it is not bullying when two students of about the same strength quarrel or fight. This definition is fairly standard and has been used over a number of years in the HBSC studies. This dimension shows a high score in QoL if these negative feelings are rare.
Financial resources	The perceived quality of the financial resources of the child/adolescent is assessed. The dimension explores whether the child/adolescent feels that he/she has enough financial resources to allow him/her to live a lifestyle which is comparable to other children/adolescents and provides the opportunity to do things together with peers.
KIDSCREEN-27 dimensions	
Physical well-being	This dimension explores the level of the child’s/adolescent’s physical activity, energy, and fitness as well as the extent to which a child or adolescent feels unwell and complains of poor health.
Psychological well-being	This dimension examines the psychological well-being of the child/adolescent including positive emotions and satisfaction with life as well as the absence of feelings such as loneliness and sadness.
Parent relations and autonomy	This dimension explores the quality of the interaction between child/adolescent and parent or carer as well as whether the child/adolescent feels loved and supported by the family. It also examines the child’s/adolescent’s perceived level of autonomy as well as the perceived quality of the financial resources of the child/adolescent.
Social support and peers	Social relations with friends and peers are considered. The dimension explores the quality of the interaction between the child/adolescent and peers as well as their perceived support.
School	This dimension explores a child’s/adolescent’s perception of his/her cognitive capacity learning and concentration and his/her feelings about school. In addition, the dimension explores the child’s view of the relationship with his/her teachers.
KIDSCREEN-10 index	
	This unidimensional measure represents a global score for the dimensions of the longer KIDSCREEN versions.

The objective of the current article is to provide an overview of the development of the KIDSCREEN, to summarize and provide examples of its extensive applications in Europe and elsewhere, and describe the development of a new computerized adaptive test (KIDS-CAT) based on KIDSCREEN experiences.

## Methods

### Development and validation of the KIDSCREEN

**Fig. 1:**
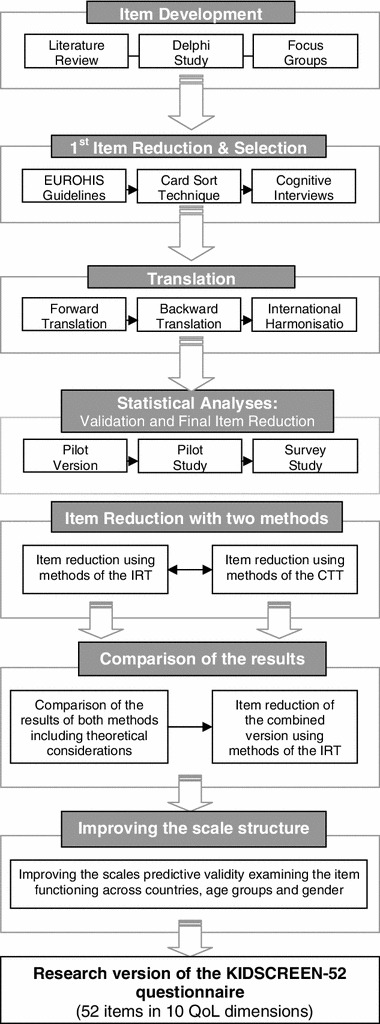
Flowchart showing development process of the KIDSCREEN tool

Conceptually, the KIDSCREEN instruments are based on the definition of QoL as a multidimensional construct covering physical, emotional, mental, social, and behavioral components of well-being and functioning as perceived by patients and/or other individuals. The KIDSCREEN project used a simultaneous approach to include 13 European countries in the cross-cultural harmonization and development of the measures. Content for the KIDSCREEN questionnaire was generated from a literature review [5], a Delphi exercise with experts in QoL measurement in children [11], and focus groups with children and parents [12]. Focus group work in the participating European countries led to the formulation of 2,505 statements which formed the original pool of possible items for the questionnaire. After an item reduction process involving redundancy rating and card sorting (Fig. 1), 179 items were selected to form the basis of a draft questionnaire for pilot testing. Administration in a pilot study with 3,019 children in seven European countries provided data which allowed for further item reduction using a combination of classical test theory (CTT) and item response theory (IRT) so as to define the final and definitive version of 52 items covering 10 dimensions of QoL [6, 13]. From this version, the KIDSCREEN-27 was produced using basic item analyses, confirmatory and explorative factor analyses, and IRT [8, 9] and the KIDSCREEN-10 was developed in turn from KIDSCREEN-27 using Rasch analysis [10].

All three KIDSCREEN questionnaires were psychometrically tested using data obtained in a multicenter European study which included a sample of 22,827 children recruited in 13 countries [14]. Participants completed the KIDSCREEN-52 together with one or more other QoL instruments for children and adolescents, such as the pediatric quality of life inventory (PedsQL) [15], Child Health and Illness Profile-Adolescent Edition (CHIP-AE—in children aged 12 years and over) [16] or the youth quality of life instrument—surveillance version (YQOL-S) [17]. The reliability and validity of the 52-, 27-, and 10-item versions of KIDSCREEN were tested primarily using a CTT approach, though Rasch analysis was also used. Test–retest reliability was assessed in approximately 10 % of the overall sample by administering the questionnaire on two occasions 2 weeks apart. The instruments’ convergent and known groups’ validity was tested by examining correlations with similar instruments and investigating whether KIDSCREEN-27 and KIDSCREEN-52 discriminated between groups defined by differences in health status. The underlying structure of the 27- and 52-item versions was examined using factor analysis and the criterion validity of KIDSCREEN-10 and KIDSCREEN-27 was analyzed by determining the magnitude of correlations with the KIDSCREEN-52. All validity testing was carried out in both the self-complete and proxy versions. Further analyses were performed to determine the cross-cultural validity of the different language versions [9]. Population norms are available at http://www.kidscreen.org.

To test responsiveness and sensitivity to change in the KIDSCREEN instruments, they have been included in longitudinal studies which provide evidence of this property. One example of such a study was a 3-year follow-up study in Spain, which investigated changes in QoL in a representative, population-based sample of children and adolescents in Spain [18] and how changes in mental health affected QoL over the same period [19]. Another example is the German longitudinal study of mental health in children and adolescents [BELLA study, 20].

## Results

### KIDSCREEN versions: content and factor structure

**Fig. 2:**
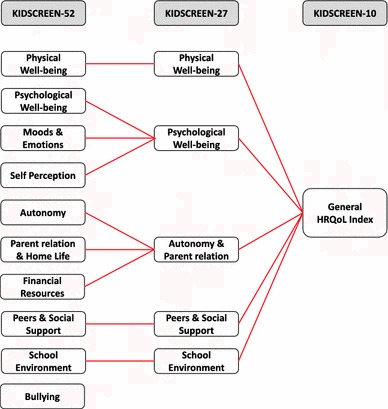
Dimension of the KIDSCREEN instruments and relationship between versions

The dimension content of the 52-, 27-, and 10-item versions is shown in Fig. 2 and Table 1. The KIDSCREEN-52 requires approximately 15 min to complete, compared to 10 min for the KIDSCREEN-27, and 5 min for the KIDSCREEN-10. The latter does not provide dimension scores, but one global score. Items in all versions are answered on 5-point Likert type scales assessing frequency or intensity. The questionnaires can be completed in person at home, in a classroom, or other settings. They can be administered by telephone, computer, in face-to-face interviews, or in mail surveys. *T*-scores and percentages are available in many languages to help with score interpretation [5].

### Factor structure and item characteristics

The results of the factor analysis with the 52-item version showed that the specified 10-dimensional structural equation model fitted the data well, with an RMSEA of 0.062 and a CFI of 0.976 (see Table 2) [7]. The model appeared to be equally satisfactory in children and adolescents. For the 27-item version, a five-factor model explained 56.9 % of the variance (RMSEA = 0.068) with a factor structure that was highly replicable in individual countries [9]. Testing of individual KIDSCREEN items using item fit statistics within the IRT partial credit model (PCM) showed that all items except one displayed an infit mean square between 0.80 and 1.20 and thus fulfilled the strong assumptions of the PCM. The PCM assumes that all items of a scale are indicators of a single unidimensional latent trait and that item–answer–characteristic curves resemble a logistic function with equal slopes [21]. Using logistic regression to test for differential item functioning (DIF) [22] across countries, age and gender groups (8 to 11 vs. 12 to 18 years) showed that only a small number of items displayed any degree of cultural DIF and qualitative examination of item content indicated that it could be attributed to the fact that those items were measuring secondary aspects which are relevant to the trait being measured but vary across the groups to be compared [23].

**Table 2:** Scaling success, Rasch measurement item fit, DIF, and CFA

	Scaling success	Rasch item fit	Country DIF	Age DIF	Gender DIF	CFA goodness of fit	
	Converge. > discrim.^a^ (%)	Infit mean square min–max^b^	∆ − *R* ^2^ min–max	∆ − *R* ^2^ min–max	∆ − *R* ^2^ min–max	RMSEA	CFI
KIDSCREEN-52						0.049	0.979
Physical well-being	100	0.887–1.126	0.006–0.041	0.001–0.004	0.001–0.001		
Psychological well-being	100	0.946–1.138	0.013–0.030	0.001–0.005	0.001–0.002		
Moods and emotions	100	0.813–1.225	0.005–0.027	0.001–0.011	0.001–0.007		
Self-perception	97.8	0.885–1.070	0.011–0.038	0.002–0.005	0.001–0.003		
Autonomy	100	0.896–1.084	0.005–0.015	0.001–0.017	0.001–0.002		
Parent relations and home life	100	0.885–1.084	0.007–0.029	0.001–0.009	0.001–0.002		
Social support and peers	100	0.801–1.264	0.014–0.042	0.001–0.004	0.001–0.004		
School environment	100	0.900–1.136	0.006–0.018	0.001–0.012	0.001–0.002		
Social acceptance (bullying)	100	0.924–1.100	0.025–0.025	0.001–0.008	0.001–0.001		
Financial resources	100	0.965–1.021	0.003–0.006	0.001–0.002	0.001–0.001		
KIDSCREEN-27						0.065	0.962
Physical well-being	100	0.887–1.126	0.006–0.041	0.001–0.004	0.001–0.001		
Psychological well-being	100	0.917–1.078	0.015–0.034	0.002–0.008	0.001–0.007		
Parents and autonomy	100	0.892–1.137	0.011–0.029	0.001–0.025	0.001–0.008		
Social support and peers	100	0.860–1.091	0.016–0.037	0.001–0.004	0.001–0.003		
School environment	100	0.937–1.038	0.006–0.018	0.001–0.005	0.001–0.002		

Range of *N* = 18533-21326

^a^ corrected item-scale correlation higher than correlation of item with other scale

^b^ 0.8–1.2 = good fit

### Scale characteristics and reliability

As shown in Table 3, the three KIDSCREEN versions generally showed excellent scale characteristics in terms of missing responses, floor and ceiling effects, and internal consistency. Cronbach’s alphas are ranging from 0.77 to 0.89 for the dimensions of the 52-item version, from 0.80 to 0.84 for the 27-item dimensions, and 0.82 for the KIDSCREEN-10. Test–retest reliability was also generally satisfactory to excellent with ICCs ranging from 0.56 to 0.77 for the 52-item version, 0.61 to 0.74 for the 27-item version, and 0.70 for the 10-item version. Only two of the dimensions in the KIDSCREEN-52 (social acceptance and financial resources) showed ceiling effects which were above the accepted threshold of 15 %.

**Table 3:** Scale description and internal consistency of KIDSCREEN child and adolescent version

	Items	*n*	Mean *T* value	SD	Missing (%)	Floor (%)	Ceiling (%)	Cronbach’s alpha	ICC
KIDSCREEN-52 dimensions									
Physical well-being	5	21,266	49.94	9.88	2.47	0.06	5.24	0.80	0.65
Psychological well-being	6	21,488	49.92	9.87	1.45	0.08	9.64	0.89	0.62
Moods and emotions	7	21,386	49.83	9.70	1.92	0.04	8.24	0.86	0.58
Self-perception	5	21,484	50.17	10.18	1.47	0.10	11.59	0.79	0.69
Autonomy	5	21,505	50.11	10.14	1.37	0.18	11.29	0.84	0.56
Parents relation	6	21,328	50.13	10.16	2.18	0.13	15.45	0.89	0.72
Social support and peers	6	21,283	49.88	9.95	2.39	0.29	7.45	0.85	0.61
School environment	6	21,299	50.05	10.14	2.63	0.19	4.90	0.87	0.77
Social acceptance (bullying)	3	21,496	50.13	10.16	1.41	0.32	49.10	0.77	0.57
Financial resources	3	21,183	50.19	10.21	2.85	1.83	24.46	0.89	0.68
KIDSCREEN-27 dimensions									
Physical well-being	5	21,266	49.94	9.88	2.47	0.06	5.24	0.80	0.65
Psychological well-being	7	21,374	49.77	9.56	1.97	0.01	5.63	0.84	0.64
Parents and autonomy	7	20,969	49.99	9.94	3.83	0.02	6.36	0.81	0.66
Peers	4	21,430	49.94	10.02	1.72	0.37	14.87	0.81	0.61
School	4	21,340	50.01	10.06	2.13	0.22	7.65	0.81	0.74
KIDSCREEN-10 index									
General QoL index	10	20,823	49.85	9.58	4.50	0	1.97	0.82	0.55

*SD* standard deviation, *ICC* intra-class correlation coefficient

### Validity

All three KIDSCREEN instruments showed good results in terms of convergent, known groups’, and criterion validity. With regard to convergent validity, correlations between other QoL questionnaires and KIDSCREEN instruments were generally moderate to high for dimensions assessing similar constructs. Examples were correlations of 0.44 between the PedsQL physical functioning dimension and the KIDSCREEN-52 physical well-being scale, 0.53 between the PedsQL emotional functioning domain and the KIDSCREEN moods and emotions dimension, or *r* = 0.60 between the KIDSCREEN physical well-being and the CHIP satisfaction domain. Similar strengths and patterns of coefficients were seen between the other QoL measures used and KIDSCREEN-27 and KIDSCREEN-10 [5].

Results of testing known group validity were also positive, with KIDSCREEN scores discriminating between groups expected to show a difference in QoL. Examples were the differences between children with and without physical and mental health problems defined by their scores on the Children with Special Health Care Needs screener (CSHCN) and the Strengths and Difficulties Questionnaire (SDQ), as shown in Table 4. Children with special health care needs reported a lower physical and psychological well-being in comparison with healthy children. The differences between both groups were significant with small to moderate effect sizes (ES). Further, Table 4 indicates that children and adolescents with mental health problems displayed significant and sizeable lower QoL values in all scales of the KIDSCREEN-52, KIDSCREEN-27, and KIDSCREEN-10 index versions. As hypothesized, the effect was highest for the KIDSCREEN dimensions psychological well-being and moods and emotions. The effect can be classified as moderate/large. Similar large effects can be found for the social acceptance (bullying) dimension of the KIDSCREEN-52 and the General QoL index. Higher KIDSCREEN scores were also seen for children in higher socioeconomic categories defined using the FAS and in younger children compared to adolescents (small to moderate ES) [24].

**Table 4:** Differences in KIDSCREEN dimension scores by health care needs (CSHCN) and mental health status (SDQ)

	Health care needs			Mental health status			
	Healthy	CSHCN (+)	Effect size^a^	Healthy	Borderline	Noticeable	Effect size^a^
	Mean *T* value (SD)	Mean *T* value (SD)		Mean *T* value (SD)	Mean *T* value (SD)	Mean *T* value (SD)	
KIDSCREEN-52							
Physical well-being	51.01 (9.77)	46.96 (10.02)	0.41	51.10 (9.75)	48.06 (9.50)	46.95 (10.74)	0.42
Psychological well-being	50.54 (9.63)	47.67 (9.84)	0.30	50.95 (9.43)	46.80 (9.97)	45.19 (10.26)	0.59
Moods and emotions	50.27 (9.40	47.78 (9.46)	0.26	50.84 (9.28)	45.82 (8.61)	43.92 (9.15)	0.73
Self-perception	50.57 (10.01)	48.84 (10.00)	0.17	50.92 (9.93)	47.39 (9.56)	46.83 (10.13)	0.41
Autonomy	50.33 (10.08	48.80 (9.87)	0.15	50.63 (9.98)	47.79 (10.14)	47.42 (10.11)	0.32
Parent relations and home life	50.05 (9.89)	47.92 (9.94)	0.21	50.51 (9.69)	46.17 (9.80)	44.96 (10.41)	0.56
Social support and peers	49.85 (9.83)	47.06 (10.06)	0.28	50.17 (9.69)	46.52 (9.74)	44.79 (10.83)	0.54
School environment	50.33 (10.07)	48.32 (10.19)	0.20	50.83 (9.98)	46.42 (9.81)	45.19 (10.04)	0.56
Social acceptance (bullying)	49.93 (9.91)	47.22 (11.10)	0.27	50.42 (9.62)	46.11 (11.03)	43.31 (11.91)	0.70
Financial resources	49.98 (10.19)	48.06 (10.55)	0.19	50.46 (10.03)	46.32 (10.14)	44.71 (11.10)	0.56
KIDSCREEN-27							
Physical well-being	51.01 (9.77)	46.96 (10.02)	0.41	51.10 (9.75)	48.06 (9.50)	46.95 (10.74)	0.42
Psychological well-being	50.29 (9.30)	47.59 (9.24)	0.29	50.77 (9.18)	46.10 (8.79)	44.46 (8.94)	0.68
Parents and autonomy	49.98 (9.80)	47.87 (9.44)	0.22	50.44 (9.71)	46.15 (8.95)	44.94 (9.00)	0.56
Social support and peers	49.83 (9.88)	47.11 (10.29)	0.27	50.18 (9.70)	46.41 (10.11)	44.68 (11.12)	0.55
School environment	50.30 (9.96)	47.86 (10.03)	0.24	50.79 (9.85)	46.09 (9.59)	44.63 (9.79)	0.62
KIDSCREEN-10							
General QoL index	50.33 (9.58)	47.38 (8.84)	0.31	50.77 (9.49)	45.98 (8.39)	44.38 (8.36)	0.67

Finally, statistically significant correlations between the 10- and 27-item KIDSCREEN scores and the majority of the KIDSCREEN-52 scales indicated satisfactory criterion validity, and KIDSCREEN-27 dimensions were found to explain 39–92 % of the variance in the corresponding KIDSCREEN-52 dimensions.

The proxy versions of the three KIDSCREEN instruments (see Table 5) also showed highly satisfactory psychometric properties [13].

**Table 5:** Scale description and internal consistency of KIDSCREEN proxy version

	Items	Mean *T* value	SD	Floor (%)	Ceiling (%)	Cronbach’s alpha	ICC
KIDSCREEN-52 dimensions							
Physical well-being	5	50.7	10.0	0.0	5.4	0.82	0.62
Psychological well-being	6	50.3	9.8	0.0	5.8	0.90	0.51
Moods and emotions	7	50.2	9.7	0.0	6.7	0.84	0.45
Self-perception	5	50.3	9.9	0.0	9.4	0.76	0.53
Autonomy	5	50.0	9.9	0.0	10.8	0.86	0.48
Parents relation	6	49.7	9.8	0.0	7.9	0.87	0.50
Social support and peers	6	49.7	10.0	0.1	3.7	0.87	0.48
School environment	6	50.1	10.0	0.0	4.4	0.88	0.62
Social acceptance (bullying)	3	49.5	9.9	0.0	45.2	0.82	0.48
Financial resources	3	49.6	10.0	1.6	16.1	0.89	0.53
KIDSCREEN-27 dimensions							
Physical well-being	5	50.7	10.0	0.0	5.4	0.80	0.61
Psychological well-being	7	50.2	9.8	0.0	2.8	0.82	0.52
Parents and autonomy	7	49.8	9.8	0.0	3.0	0.78	0.51
Peers	4	49.6	10.0	0.2	5.4	0.84	0.44
School	4	50.1	9.9	0.0	6.0	0.83	0.60
KIDSCREEN-10 index							
General QoL index	10	50.2	10.0	0.0	0.8	0.78	0.56

*SD* standard deviation, *ICC* intra-class correlation coefficient

### Results from longitudinal studies: evidence of responsiveness?

In the Spanish KIDSCREEN follow-up study [18, 19], response rate at 3-year follow-up was 54 % and QoL was observed to have worsened in eight out of the ten KIDSCREEN dimensions, with effect sizes ranging from −0.10 (moods and emotions) to −0.34 (psychological well-being). However, when the sample was stratified by age group and gender, effect sizes ranging from 0.48 social acceptance (bullying) to −0.60 (psychological well-being) for boys and 0.33 social acceptance (bullying) to −0.56 (psychological well-being) for girls were observed, indicating moderate effect sizes. The KIDSCREEN-52 did therefore seem to be capable of reflecting change over time in this sample. Worsening of QoL was attributed at least in part to the onset of puberty. Additional analysis from this study found that changes in mental health status measured using the SDQ were also associated with changes on KIDSCREEN-52; respondents who worsened on the SDQ showed the greatest deterioration, particularly on the dimension of psychological well-being (ES = −0.81), a finding which provides evidence of the instrument’s longitudinal validity.

## Adaptation into other languages

Although content for the questionnaire was generated simultaneously through focus groups in several countries, the source version of each item was created in English. It was therefore necessary to translate those items into the relevant target languages. This was done using a standardized methodology based on international cross-cultural translation guidelines [25, 26]. The first step involved a forward–back–forward translation technique in which the original English draft was translated by two translators working independently. After reconciliation, a consensus version was back translated into English and compared to the original. This led to a second consensus version in each language. These were harmonized cross-culturally in an international telephone conference and a pretest version was evaluated in cognitive debriefing interviews. A similar procedure has been used to produce any new language versions of the instrument developed since the original project was completed. Currently, the self-complete child–adolescent version has been translated into 38 languages in Europe, North America and South America, Africa and Asia, and the proxy version into 33 languages (see Table 6), including the languages in the original development process.

**Table 6:** KIDSCREEN available country/language versions

Child/adolescent version			Countries	Proxy version		
10	27	52		10	27	52
✓	✓	✓	Argentina	✓	✓	✓
✓	✓	✓	Austria	✓	✓	✓
✓	✓	✓	Australia	✓	✓	✓
✓	✓	✓	Belgium	–	–	–
✓	✓	✓	Brazil	✓	✓	✓
✓	✓	✓	Chile	✓	✓	✓
✓	✓	✓	Colombia	–	–	–
✓	✓	✓	Croatia	✓	✓	✓
✓	✓	✓	Czech Republic	✓	✓	✓
✓	✓	✓	Denmark	✓	✓	✓
✓	✓	–	Finland	✓	✓	
✓	✓	✓	France	✓	✓	✓
✓	✓	✓	Germany	✓	✓	✓
✓	✓	✓	Greece	✓	✓	✓
✓	✓	✓	Hungary	✓	✓	✓
✓	✓	✓	Iran	✓	✓	✓
✓	✓	✓	Ireland	✓	✓	✓
✓	✓	✓	Italy	✓	✓	✓
✓	✓	✓	Iceland	✓	✓	✓
✓	✓	✓	Japan	✓	✓	✓
✓	✓	✓	Korea	✓	✓	✓
✓	✓	✓	Kenya (Dholuo)	✓	✓	✓
✓	✓	✓	Mexico	–	–	–
✓	✓	✓	The Netherlands	✓	✓	✓
✓	✓	✓	Norway	✓	✓	✓
✓	✓	✓	Poland	✓	✓	✓
✓	✓	✓	Portugal	✓	✓	✓
✓	✓		Romania	✓	✓	–
✓	✓	✓	Russia	–	–	–
✓	✓	✓	Serbia	✓	✓	✓
✓	–	–	Slovenia	–	–	–
✓	✓	✓	Spain	✓	✓	✓
✓	✓	✓	Sweden	✓	✓	✓
✓	✓	✓	Switzerland	✓	✓	✓
✓	✓	✓	Uganda (Luganda)	✓	✓	✓
✓	✓	✓	United Kingdom	✓	✓	✓
✓	✓	✓	USA	✓	✓	✓
✓	✓	✓	Venezuela	✓	✓	✓

## Applications

Between 2005 and 2012, the KIDSCREEN instruments have been used in 49 mostly clinical and epidemiological studies. The measurements have been applied predominantly in European countries, but also beyond for example in Korea, Colombia, Uganda, and Kenya.

Details of three of the largest and most relevant international studies in which KIDSCREEN instruments have been utilized to date are described below:

### Health behavior in school-aged children (HBSC) study

The KIDSCREEN-10 index was included from 2005 on as a measure for positive well-being in the “Health Behavior in School-Aged Children” (HBSC) study [27, 28] which is conducted in collaboration with the WHO Regional Office for Europe. The aim of these studies, which are repeated periodically, is to increase understanding of young people’s health and well-being and, more specifically, to gain insight into health behaviors and their social context. The 2005/2006 HBSC survey took place in 41 European and North-American countries and Israel and included children aged 11, 13, and 15 years attending regular schools. Interviewers or teachers distributed the study questionnaire in class and more than 200,000 children filled in the study questionnaires and returned them in anonymous envelopes. Fifteen countries included the KIDSCREEN-10 as an optional package and the instrument was completed by 78,383 children and adolescents (51 % female). National samples were representative of school-aged children attending regular schools. Mean values for the school-aged children varied from 41.2 (Turkey) to 50.7 (Austria) [29].

### Eurobarometer study

This study, the Flash Eurobarometer (No 246) on “Parents’ views on the mental health of their child,” used the KIDSCREEN-10 indicator on quality of life and mental well-being to assess parents’ reports of their children’s health and well-being between and within the 27 member states of the European Union. The study was conducted by Eurobarometer for the European Commission, Health and Consumers Directorate General [30]. Overall, 12,783 telephone interviews were conducted with parents of children 6 to 17 years old in the EU27 States. Parents reported children’s QoL on the Rasch-scaled KIDSCREEN-10 as well as their occupational status and education level. Multilevel and regression analyses were used to determine the effect of parental occupation and education level, as well as gross domestic product per capita and income inequality, on KIDSCREEN-10 scores. Low QoL was reported in 11.6 % of cases with cross-national variation accounting for 13 % of the total variance in QoL. Higher national wealth and lower income inequality all over Europe were associated with better population QoL and explained 13.5 % of the country differences. Older age of the child [OR = 2.2/2.0 (boys/girls)] and a medium (OR = 1.2) or low (OR = 1.4) occupational status of the parent were associated with a higher risk of lower QoL. Low educational status in European countries also increased the risk for low QoL in children (OR = 1.3).

### The Sparcle study: using KIDSCREEN-52 to measure QoL in cerebral palsy

This European study was designed to assess the self-reported QoL of children with cerebral palsy, as well as to explore the factors influencing it, and how it compared with QoL in the general population. They used the KIDSCREEN-52 child and proxy versions to assess QoL. A total of 1,174 children aged 8 to 12 years were randomly selected from eight population-based registers of children with cerebral palsy in six European countries and 743 (63 %) agreed to participate; one further region recruited 75 children from multiple sources. About 61 % of those who agreed to participate were able to self-complete the KIDSCREEN-52, while 318 (39 %) with severe intellectual impairment could not self-report. Multivariate regression was used to relate QoL to impairments, pain, and sociodemographic characteristics. Comparisons were made with QoL data from the general population in the 5 countries in which that information was available. The study showed that impairments were significantly associated with KIDSCREEN domains; severely limited self-mobility was significantly associated with reduced physical well-being, intellectual impairment with reduced mean for moods and emotions and autonomy, and speech difficulties with poorer relationships with parents. Pain was common and associated with lower QoL on all domains. Impairments and pain explained up to 3 and 7 %, respectively, of the variation in QoL. On the other hand, children with cerebral palsy had similar QoL to children in the general population in all domains except schooling, in which evidence was equivocal, and physical well-being, in which comparison was not possible [31, 32].

## New initiatives

The most recent advance within the KIDSCREEN project is the development of a computer-adaptive test (CAT) version—the KIDS-CAT. A computer version to fill out the questionnaire via computer, being computer assisted, not computer adaptive, already existed. A particular aim of this CAT-initiative is to accelerate the use of pediatric QoL measurement in healthy children and routine clinical practice, an area in which patient-reported outcomes (PROs) are still underused [33, 34]. Currently, there are no German CAT tools to assess pediatric QoL in an efficient and precise way. The new initiative is funded by the German federal ministry of Education and Research from 2012 to 2015 (Title of project: Quality of Life in Chronically Ill Children: Development and Validation of Computer-Adaptive Testing in Routine Pediatric Care, Contract No: 01GY1111) and uses the experience gained with KIDSCREEN to create a CAT version [35], which will allow efficient, short, highly precise, and easily assessed QoL measurement in children and adolescents via computer technology. The first application will be available in 2013 as software for computers and via the Internet.

KIDS-CAT has been developed by applying a combination of CTT and IRT methods [36, 37] and is analogous to the methods used by the US-wide patient-reported outcome initiative (PROMIS) [38, 39]. The KIDS-CAT content is based on the KIDSCREEN-27 domain structure, and item banks include all KIDSCREEN items plus items used in other established pediatric health surveys administered in large-scale German, Swiss, and Austrian studies (*n* = 10,000–20,000 children/adolescents).

The item banks were developed by analyzing data from 10,577 to 19,392 children/adolescents (per domain). Item generation was performed in 6 iterative steps: (1) item review of all survey items; (2) a Delphi process by six QoL experts to determine the item contents fitting the five KIDS-CAT dimensions; (3) confirmatory factor analyses (CFA) to test the unidimensionality of the item banks; (4) analyses of DIF by age, sex, ethnic group, education, and sociodemographic background; (5) item response curves (IRC) analyses to determine response option functioning; and (6) item parameter estimation.

A total of 162 items were selected from an initial item pool of 377 items. Those selected showed the highest levels of content validity, had factor loadings of >.4 and residual correlations <.25, had no DIF (*R* ^2^ < 5 % and *p* < 0.001), displayed monotonic and chronologically ordered response option curves, and allowed item calibration. The final KIDS-CAT instrument contains five item banks covering the psychological (46 items), physical (26 items), family (26 items), peer (26 items), and school well-being (31 items) domains. The calibrated item banks were used as the basis for a KIDS-CAT pilot version, which was programmed using C++ by IT experts. A designer team created a child-friendly front end in close collaboration with the experts which was tested in focus groups with children.

Currently, the KIDS-CAT is being implemented in a longitudinal study in 1,200 school children and 300 chronically ill children to assess its feasibility, reliability, validity, and responsiveness to change. It should also help to determine equivalence with the paper version as well as providing normed data for healthy children and chronically ill children with asthma or diabetes. During the longitudinal study, healthy children will respond to the KIDS-CAT at baseline, 6 months, and 1 year, while chronically ill children will respond to KIDS-CAT every month for a year. Data collection in the chronically ill sample also includes the longitudinal assessment of health/disease status by children, parents, and clinicians at baseline, 6 months, and 1 year. The study aims to investigate the feasibility of the tool as a screening and monitoring instrument in healthy children and in routine clinical practice. While all KIDS-CATs will be administered via the Internet in this study, future studies will explore applications on cell phones and tablet devices.

## Discussion

The KIDSCREEN instrument is a generic measure of QoL which is suitable for use as a screening, monitoring, and evaluation tool. The availability of three different versions makes it an adaptable tool which can be used in many different settings, including clinical environments, schools, or the respondent’s home. It can be administered by professionals in the fields of public health, epidemiology, and medicine. It can be used in healthy and chronically ill children and adolescents from 8 to 18 years and can be self-completed or administered through a proxy version for parents or primary caregivers. The internationally developed KIDSCREEN Quality of Life Questionnaire comprehensively assesses physical, psychological, social, family, and school aspects of well-being and the functional ability of children and adolescents.

One very important step in the development of the instrument was to ask children and adolescents in group discussions about their understanding of the concepts of health and well-being. Their opinions and beliefs served as a basis for the instrument and the resulting items reflect their experiences and lifestyle. Children’s understanding of the items and their acceptability were evaluated in several phases of instrument development. An additional advantage of the instrument is that it was developed simultaneously in several European countries and contains country specific as well as multicultural aspects. The development process was also very thorough, and a range of psychometric approaches was applied in item development and testing, including common and advanced psychometric analyses such as IRT and structural equation modeling (SEM). A manual with detailed information about psychometric properties, scoring instructions, and interpretation of test scores as well as international and national norm data is available for the KIDSCREEN instrument and can be retrieved from http://www.kidscreen.org.

The instrument’s excellent psychometric properties based on the data from a sample of 22,827 children and adolescents from 13 European countries [6] likely reflect the rigorous development process. In contrast to other widely used generic pediatric quality of life measures like PedsQL [15], CHIP [16], or CHQ [40], the KIDSCREEN offers the following advantages and differences. First, the KIDSCREEN was developed simultaneously in 13 countries. Therefore, in comparison with all other QoL measures, the KIDSCREEN instruments are truly cross-national. Second, the KIDSCREEN includes a modern IRT-based approach, which has not been applied to other measurements. Third, the KIDSCREEN was developed in close collaboration with the DISABKIDS [4], which covers disease-specific QoL in children and adolescents with chronic conditions, ensuring a similar and complementing disease-specific measurement. Disease-specific complementing versions are also available for the PedsQL but not for CHIP or CHQ. Fourth, KIDSCREEN offers three versions of different length, which can be used according to content and setting. Most other questionnaires are available only in one version of length. Fifth, the KIDSCREEN-10 index is well applicable in routine monitoring and screening and helps to reduce response burden. Further, like the EQ-5D-Y [41], the KIDSCREEN index can be used for cost-utility analyses, which is important in health economic studies. Challenges in using the KIDSCREEN include assessing children younger than 8 years. This gap is closed by the PedsQL offering scales to be used for infants [42].

From 2009, 695 researchers and clinicians officially registered to use the KIDSCREEN and gave very positive feedback regarding its feasibility. Further, the KIDSCREEN measures are used to contribute to European policies by providing information about the types and distribution of quality of life impairments (nationally as well as Europe-wide). They aim at improving how children and adolescents perceive their health status, thus helping to identify populations at risk. The cross-cultural development of the instrument and therefore the lack of cultural DIF should make it possible to compare and contrast results from different countries, at least within the European context.

Finally, it is to be hoped that the new KIDS-CAT initiative will provide greater measurement precision coupled with a lower test burden (at an expected application of 5–6 items per domain), thereby reducing the administrative burden for respondents and for clinicians. If that is the case, it is hoped that this new technology will accelerate the implementation of patient-reported outcome measures in routine care. This in turn could help to optimize communication between clinicians and the child/adolescent and his/her parent as well as identifying areas of well-being and functioning in which improvements are possible.
